# Transcriptome sequencing of *Salvia miltiorrhiza* after infection by its endophytic fungi and identification of genes related to tanshinone biosynthesis

**DOI:** 10.1080/13880209.2019.1680706

**Published:** 2019-11-07

**Authors:** Yan Jiang, Lei Wang, Shaorong Lu, Yizhe Xue, Xiying Wei, Juan Lu, Yanyan Zhang

**Affiliations:** Key Laboratory of the Ministry of Education for Medicinal Resources and Natural Pharmaceutical Chemistry, National Engineering Laboratory for Resource Development of Endangered Crude Drugs in Northwest of China, Shaanxi Normal University, Xi’an, China

**Keywords:** Interaction, metabolic production, plant stress, MVA pathway, MEP pathway, DEGs

## Abstract

**Context:** *Salvia miltiorrhiza* Bunge (Labiatae) is a traditional Chinese herb. Endophytic fungi, which are biotic elicitors, can induce accumulation of secondary metabolites in their host plants.

**Objective:** To analyze the interaction mechanism between *S. miltiorrhiza* and endophytic fungi.

**Materials and methods:** Endophytic fungi U104 producing tanshinone IIA were isolated from the healthy disease-free tissue of root of *S. miltiorrhiza* by conventional methods. The endophytic fungus U104 of *S. miltiorrhiza* was co-cultured with the sterile seedlings of *S. miltiorrhiza* for 20 d (temp:day/night = 26 °C/18 °C, photoperiod:12/12 h, illuminance:2000 Lx). Transcriptome sequencing of *S. miltiorrhiza* seedlings after 20 d of co-cultivation was performed using the Illumina platform.

**Results:** A total of 3713 differentially expressed genes (DEGs) were obtained. These different expression genes, such as STPII, LTP2, MYB transcription factors, CNGC, CDPK, Rboh, CaM, MAP2K1/MEK1, WRKY33, SGT1/SGT and Hsp90/htpG, showed that host *S. miltiorrhiza* had biological defence response in the initial stage of interaction. Under the induction of endophytic fungi, 14 key enzyme genes were up-regulated in the tanshinone biosynthesis pathway: DXS, DXS2, DXR, HMGR3, AACT, MK, PMK, GGPPS2, GPPS, KSL, IDI, IPII, FDPS and CPS.

**Discussion and conclusions:** A total of 14 key genes were obtained from the tanshinone component synthesis and metabolic pathways, providing a reasonable explanation for the accumulation of tanshinone components, an accumulation induced by endophytic fungi, in the host plants. The large amounts of data generated in this study provide a strong and powerful platform for future functional and molecular studies of interactions between host plants and their endophytic fungi.

## Introduction

As a traditional Chinese herb, *Salvia miltiorrhiza* Bunge (Labiatae), has more than 1000 years of application history. Its active medicinal ingredients mainly include two groups: hydrophilic phenolic acids and lipid-soluble tanshinones. The phenolic acids possess various bioactivities including antioxidant, anti-inflammatory, anticancer, antibacterial, antivirus, antifibrotic activities (Shi et al. 2019). Phenolic acids such as salvianolic acid B (Li et al. 2014; Jing et al. 2016) and salvianolic acid A (Pan et al. 2014; Ding et al. 2016) have antitumor and antioxidant activities. Tanshinones have antitumor (Dong et al. 2011) and anti-inflammation (Ma et al. 2016) activities; cryptotanshinone can prevent and treat atherosclerosis (Suh et al. 2006), tanshinone IIA can be used for treating osteoporosis and reducing blood lipids (Kwak et al. 2006) and cholesterol content (Chen et al. 2016). Tanshinone IIA is a major lipid-soluble compound having promising health benefits. *In vivo* and *in vitro* studies showed that the tanshinone IIA and salvianolate have a wide range of cardiovascular and other pharmacological effects, including antioxidative, anti-inflammatory, endothelial protective, myocardial protective, anticoagulation, vasodilation and anti-atherosclerosis, as well as significantly reducing proliferation and migration of vascular smooth muscle cells (Ren et al. 2019). *S. miltiorrhiza* has many biological activities and excellent prospects. The majority of studies focus on using genetic engineering methods to regulate the expression of key genes in secondary metabolic pathways to directly influence the accumulation of end products. Studies have shown that overexpression of *SmGGPPS* and *SmDXSII* in hairy roots produces higher levels of tanshinone than control and single-gene transformed lines (Shi et al. 2016). Two elicitor treatments suggested that tanshinone accumulation positively correlated to the expression of key genes such as *SmGGPPS, SmCPS* and *SmKSL* (Hao et al. 2015); In the hairy roots of *S. miltiorrhiza*, overexpression of *SmERF1L1* significantly increased tanshinones production by comprehensively upregulating tanshinone biosynthetic pathway genes (Huang et al. 2019), silencing of *SmERF115* reduced the phenolic acid level, but increased tanshinone content (Sun et al. 2019). In addition, elicitors such as methyl jasmonate, salicylic acid, heavy metal ions (Co^2+^, Ag^+^ and Cd^2+^), sorbitol and ultraviolet can be used to increase the accumulation of tanshinones and phenolic acids in *S. miltiorrhiza* hairy roots (Zhao et al. 2010; Xing et al. 2014; Wang et al. 2016), whereas, biotic elicitors such as yeast extracts can also stimulate its hairy roots to produce more tanshinones (Shi et al. 2007; Wu et al. 2008).

Plant endophytic fungi refer to fungi that live inside the various tissues and organs of healthy plants during certain stages or all stages of their life cycle without causing apparent symptoms of infection in host plants. During the long-term evolutionary process, endophytic fungi are important components in medicinal plants. They form a stable and mutually beneficial symbiotic relationship with medicinal plants and can produce the same, or similar, secondary metabolites in the host plants (Venugopalan and Srivastava 2015). Endophytic fungi can also act as elicitors to rapidly activate specific genes in the secondary metabolic pathway in medicinal plants to accumulate a large amount of active ingredients (Zhai et al. 2017).

RNA sequencing (RNA-Seq) is the high-throughput sequencing of mRNA in a species. Its resolution has the accuracy of a single nucleotide, it can dynamically reflect gene transcription levels and it provides specific sequence-structure information of transcripts in samples (Hansen et al. 2011). Currently, RNA-Seq is being extensively applied in all fields, including basic biological research, medical research and drug development (Kawahara et al. 2012; Foth et al. 2014; Zhang et al. 2014). This study performed RNA-Seq on sterile plantlets of *S. miltiorrhiza* and endophytic fungi to examine the differential gene expression after infection of tissue-cultured plantlets with endophytic fungi, to understand the underlying molecular mechanism of interaction, then analyzed to provide new ideas and methods for studying the regulation of secondary metabolism in medicinal plants.

In the laboratory, two endophytic fungi producing tanshinones were isolated from the roots of *S. miltiorrhiza*: TR21 and U104. TR21 is a wild strain isolated from *S. miltiorrhiza* and U104 was induced by TR21. The strains of TR21 and U104 were identified as Ascomycota, Eurotiomycetes, Eurotiales, Trichocomaceae, *Eurotium*, *Emericella foeniculicola* Udag (Trichocomaceae). In the early stage of the experiment, the endophytic fungi TR21 and U104 of *S. miltiorrhiza* were co-cultured with the sterile *S. miltiorrhiza* seedlings for 10 d and 20 d, respectively, and the content of tanshinone in the plants was determined. The accumulation amount of tanshinone was used as the index to screen the best inducible strain and time. The results showed that the content of tanshinone in aseptic tissue culture seedlings of *S. miltiorrhiza* was 103.89, 151.08 and 155.56 µg/mL, respectively, after 10 d of co-culture with endophytic fungi TR21 and U104. After a total of 20 d of culture, the contents of tanshinone in the control, TR21 and U104 groups were 306.42, 360.51 and 444.44 µg/mL, respectively. The content of tanshinone increased to different degrees. The effect of 20 d interaction was significantly better than that of 10 d interaction, and in the process of 20 d interaction, the induction effect of U104 strain was significantly higher than that of TR21 strain. Taking the content of tanshinone as an indicator, it was found that when the sterile seedling of *S. miltiorrhiza* was treated with U104 strain for 20 days, the induction effect was the best.

## Materials and methods

### Endophytic fungus and host plant materials

**Figure 1.**
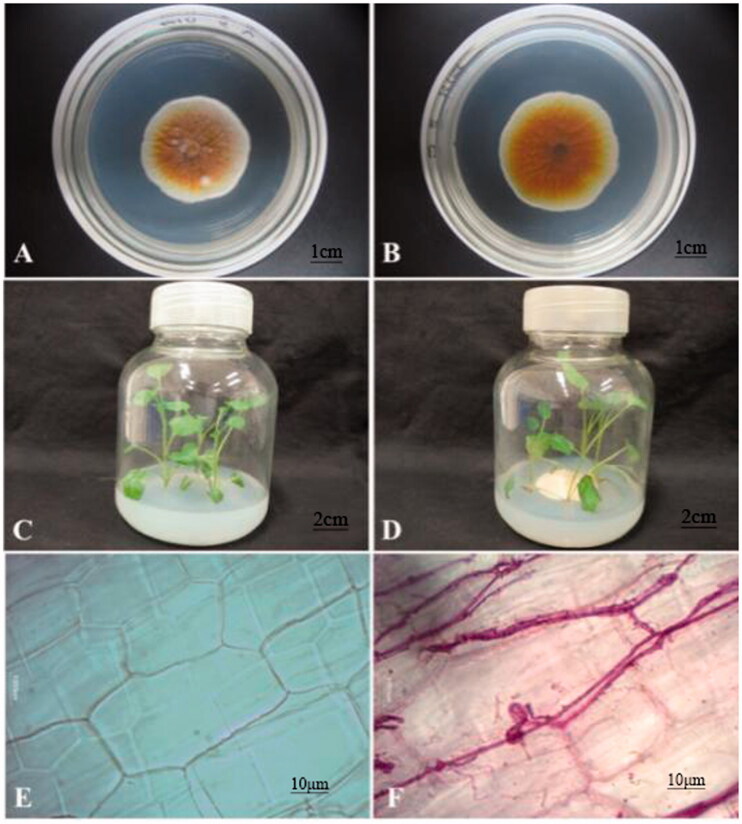
Endophytic fungus and host plant materials. (A) Positive side of U104 strain; (B) Back of U104 strain; (C) Tissue culture seedling control group; (D) Tissue culture seedling U104 group; (E) Plant tissue cell control group; (F) Plant tissue cell U104 group. A and B are test strain U104. C is just *Salvia miltiorrhiza* sterile plantlet, which is used as the control group. D is endophytic fungus and *Salvia miltiorrhiza* interaction group. E and F are the results of fungus U104 infection the roots of *Salvia miltiorrhiza* sterile plantlet tablet dyeing.

*S. miltiorrhiza* sterile plantlets were obtained by tissue culture and identified by Professor Zhezhi Wang of Shaanxi Normal University, Xi'an, Shaanxi, China. *S. miltiorrhiza* sterile plantlets that had been preserved in the laboratory and cultured for one week were used as the materials. The test strain U104 is an endophytic fungus, which was isolated from *S. miltiorrhiza* and preserved in our laboratory. The stem segments of sterile plantlets with opposite leaves were inserted in a triangular configuration directly into culture flasks containing 100 mL of MS culture medium, with three plants in each flask. The day/night culture temperature was 26 °C/18 °C, the light cycle was 12 h, and the luminance was 2000 l×. The U104 strain was inoculated into potato dextrose agar (PDA) culture medium and cultured at 28 °C for 7 d. The sterile plantlets (which had been cultured for 7 d, were 2–3 cm in height and showed consistent development status) were divided into two groups. In one group, one piece of fungal disc with a diameter of 3 ± 0.1 mm was inoculated into culture medium at 1–2 cm from the plantlets. In the other group, sterile plantlets without inoculation with fungi were used as the controls. The whole plant was taken as a sample in this study. After 10 d and 20 d of co-culture, the samples were immediately frozen in liquid nitrogen and stored in a −80 °C freezer for future use. All interaction experiments had two biological replicates. The results are shown in Figure 1.

### RNA extraction and mRNA-Seq library construction and sequencing

Total RNA in samples from two groups was extracted using a MiniBEST Plant RNA Extraction kit (TaKaRa, Dalian). After RNA samples were qualified using electrophoresis, NanoDrop, Qubit 2.0 and Agilent 2100 analyses, the cDNA library was constructed by Biomarker Technology Co., Ltd (Beijing, China). Paired-end sequencing was performed using an Illumina HiSeq 2000 sequencer and the read length of sequencing was determined with PE150.

### Sequence processing and unigene library

After the sequencing was complete, the linker sequences and low-quality reads of raw data were removed to obtain high-quality clean data. Trinity software (Grabherr et al. 2011) and paired-end method was used for sequence assembly. BLAST software (Altschul et al. 1997) was used to compare unigene sequences in the NR, Swiss-Prot (Apweiler et al. 2004), GO (Ashburner et al. 2000), COG (Tatusov et al. 2000), KOG (Koonin et al. 2004), KEGG (Kanehisa et al. 2004) and Pfam databases (Finn et al. 2014) to obtain the annotation information of the unigenes. BLAST parameter E-value is not greater than 10^−5^. The differential expression among samples was analyzed using DESeq (Anders and Huber 2010). A false discovery rate (FDR) less than 0.01 and a fold change (FC: the ratio between base mean value of the treatment group and base mean value of the blank group) no less than 2 were used as the screening standards. In addition, *r*^2^ (Schulze et al. 2012) was used as the evaluation indicator for the correlation between the biological replicates.

### Quantitative real-time RT-PCR

Fluorescence quantitative polymerase chain reaction (PCR) was performed using SYBR® Premix Ex Taq™II (Tli RNaseH Plus). The amplification system included 1 µL of cDNA (10×), 0.5 µL of each of the 10 µmol/L upstream and downstream gene-specific primers and 10 µL of 2 × SYBR Premix Ex Taq^TM^, with the total volume brought to 20 µL using ddH_2_O. The amplification condition list is shown in Table 1. Each sample had three technical replicates and three biological replicates. Data analysis was performed using the 2^−ΔΔCt^ method.

**Table 1.** Procedure of real-time PCR.

Reaction temperature	Reaction time
95 °C	30 s
95 °C	5 s (40 cycles) 30 s
primer annealing temperature	
65 °C	Melt chain curve
95 °C	From 65 °C to 95 °C (per 0.5 °C reaction 5 s)

## Results

### RNA-Seq analysis of transcriptome samples

**Figure 2.**
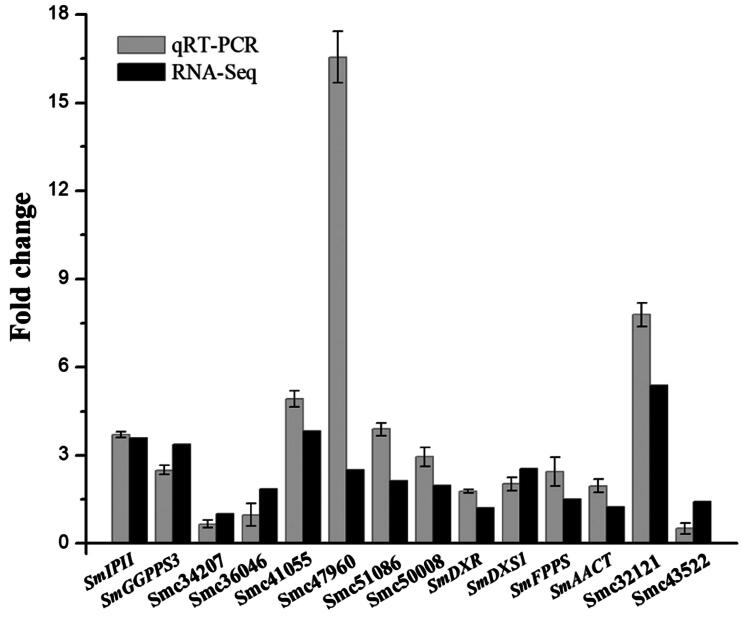
The result comparison between qRT-PCR and RNA-Seq. The expression of Smc34207, Smc36046 and Smc43522 genes was down-regulated, and the remaining genes were up-regulated, which was consistent with the RNA-Seq results.

After data filtering and quality-control analysis of the raw data, a total of 22.73 Gb clean data were obtained. At least 5.42 Gb clean data were attained for every sample. The percentages of Q30 bases in all samples were no less than 91.46% (Table 2). Sequence assembly was performed and a total of 200,043 transcripts and 96,802 unigenes were obtained, and 18,364 unigenes had lengths longer than 1 kb. Through the comparison of seven major databases, a total of 48,621 unigenes with annotation information were ultimately obtained. Based on these results, 3713 differentially expressed genes (DEGs) were obtained using the differential expression analyses. A total of 3451 genes showed up-regulated expression and the FC was more obvious at 5- to 10-fold. A total of 262 genes showed down-regulated expression, and the FC was generally between 1- and 5-fold. The scatter plot of the gene correlation of the samples showed that the expression trends of most genes in the samples of the two biological replicates were similar, and the correlation between the replicates was high (both r^2^ > 0.90). Actin was used as the internal control gene and the relative expression levels of all genes obtained using qRT-PCR were compared and validated with the FC values in the RNA-Seq results (Figure 2). The upstream and downstream gene-specific primers used in qRT-PCR are shown in Table 3. The results were basically consistent, indicating the reliability of the RNA-Seq results.

**Table 2.** The assessment table of sample sequencing data.

Samples	Library Type	Read Number	Base Number	GC Content	%≥Q30
YP1	Paired-end	20,467,209	6,116,941,620	49.88%	91.70%
YP2	Paired-end	18,122,156	5,415,384,438	49.64%	92.29%
YP1 2	Paired-end	19,083,640	5,705,066,840	50.19%	91.52%
YP2 2	Paired-end	18,362,814	5,492,791,266	51.22%	91.46%

**Table 3.** Primer sequences used for qRT-PCR amplification.

Gene	Primer sequences (5′-3′)	
	Forward	Reverse
SmActin	AGGAACCACCGATCCAGACA	GGTGCCCTGAGGTCCTGTT
SmDXR	TGTAGTCACAGGAATTGTTGGATG	GCAAGAGGAAGGACGAAAGT
SmDXS1	TGAGAGCGACTACGACTGCTTTGG	CCCATCCAGATTGGCAGTAGC
SmFPPS	TCCAGGGCCTTTACAACCAGC	TTCATCGCCGCATTGTTCAGT
SmIPI1	AGCGTGCATCCAAATCCAGAC	GATAGCTTCAAGCCCCCCTCA
SmGGPPS3	GGCCAGTGCTCTGCTGTCTGTG	TCGGCCACCTCCATCGCTT
SmAACT	ATGCTGAAGGACGGACTCTGGGAG	TTGTCAACAATGGTGGATGG
Smc32121	TGAGCAGAAACGGCAAAC	TGGGCGAGGGAGTATGA
Smc34207	GAGGAGGATGGTGTCGTA	CTTGCCAGTGAGTCTTGAT
Smc36046	CACCTCACTAACCATACTACA	TACCTGGCGTTGGATAGA
Smc41055	CTTCGTGGTGTCTAATGTTG	CCGCATCGTCTCTTGAAT
Smc47960	ACGCTCATCACTCCAACATC	CTTACCTTGACCCGAACCA
Smc51086	TTGAGGTTGGCACAGTAGGAGG	GGCGACAATGGTGGCTAAGAG
Smc50008	GTTGGACACCTTGAAGTATG	AAGACTATGCGAACATCAGA
Smc43522	ATCGGCATTCCACAGACT	CTTACATCCTCCACACCAAT

### Functional annotation and enrichment analysis of the DEGs

The annotation of the unigenes in the three GO categories showed that nodes with more obvious differences might be associated with the observed differential expression. GO enrichment analysis on unigenes with differential expression showed that the DEGs involved in the oxidation-reduction process accounted for most. Additionally, 99 genes were involved in oxidoreductase activity. Because the late stage of biosynthesis of tanshinone components involves a large amount of oxidation-reduction reactions, these genes could be used as potential candidate genes of key enzymes in the biosynthesis pathway of tanshinone components. In the cellular components, in addition to those undergoing significant enrichment in the cell nuclei, cytoplasm and ribosomes, genes enriched in the integral components of the membrane and the extracellular region also accounted for a large quantity, this might be associated with recognition proteins (receptor proteins) on the cell surface during interactions between endophytic fungi and the host plant, *S. miltiorrhiza*. In addition, a fair number of DEGs showed significant enrichment in some transcription and regulatory factors. The significance of this discovery guided our studies on signal transduction during the interaction. The KEGG pathways showed enrichment of DEGs mainly included primary metabolic processes, such as amino acid metabolism, glucose metabolism, lipid metabolism and carbon fixation, as well as secondary metabolic processes, such as phenylalanine metabolism (ko00360), terpenoid backbone biosynthesis (ko00900) and phenylpropanoid biosynthesis (ko00940). In addition, plant-pathogen interactions (ko04626) and plant hormone signal transduction (ko04075) also showed significant enrichment.

### Responsive expression of the host plant at the initial stage of induction

**Figure 3.**
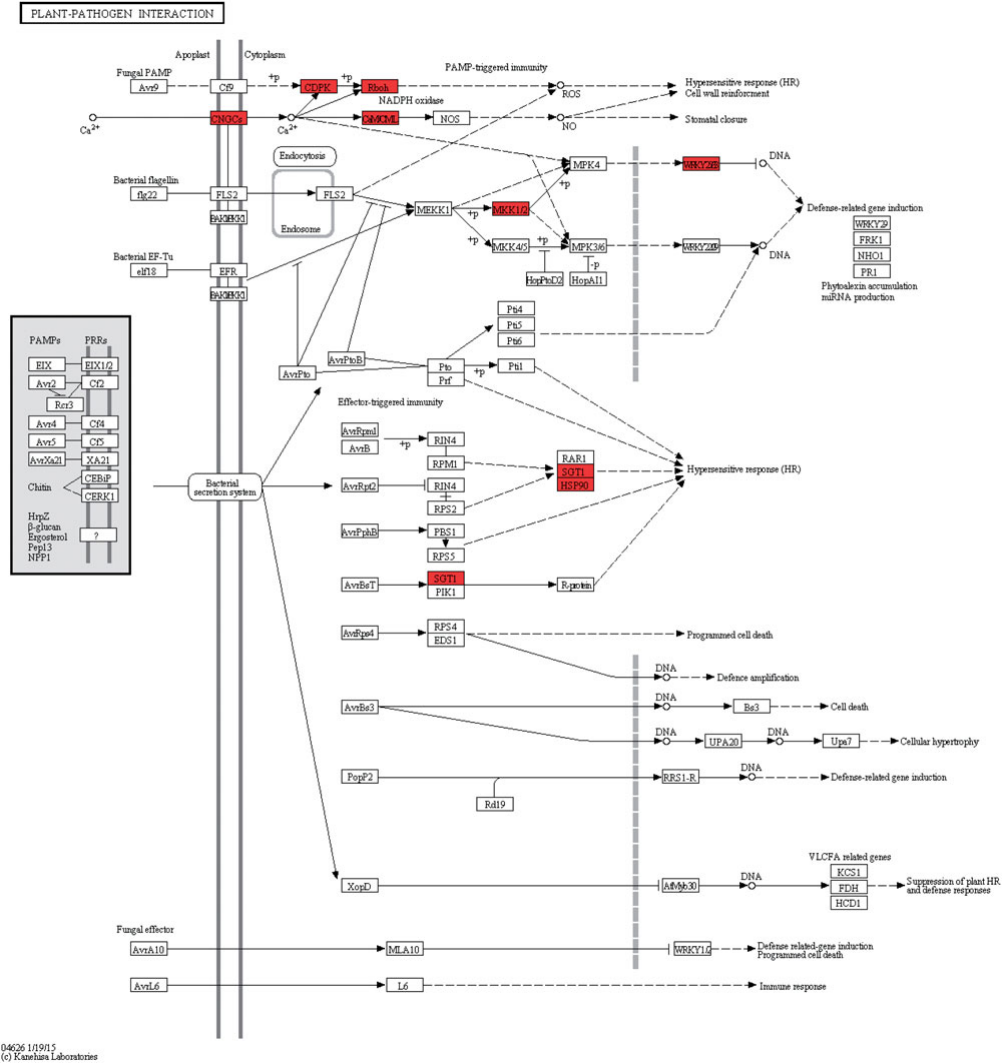
Plant-pathogen interaction. The red box represents up-regulated expression gene. When plants are stimulated by external biological stimuli, CNGCs channel opens and Ca^2+^ influx occurs. Then the activation of calmodulin (CaM) feedback inhibits the activity of CNGCs and prevents the intracellular Ca^2+^ concentration from soaring. CDPK was activated to phosphorylate downstream target proteins, MAPK was activated and WRKY transcription factors were phosphorylated. It also activates NADPH, oxidizes Rboh, and causes hypersensitivity (HR).

In the response to the induction process (Figure 3), which was induced by the endophytic fungi, in the host *S. miltiorrhiza*, eight differential genes, including CNGC (cyclic nucleotide-gated channel), CDPK (calcium-dependent protein kinase [EC:2.7.11.1]), Rboh (respiratory burst oxidase), CaM (calmodulin), WRKY33 (WRKY transcription factor), MAP2K1/MEK1 (mitogen-activated protein kinase kinase 1 [EC:2.7.12.2]), SUGT1/SGT1 (suppressor of G2 allele of SKP1) and HSP90A/htpG (molecular chaperone HtpG), all showed upregulated expression. CNGC is a non-selective cation channel and is a component of the signal transduction pathway in plant systems (Jha et al. 2016). When plant is induced by its endophytic fungi, the CNGC channels will open and Ca^2+^ influx occurs (Verret et al. 2010; Ma 2011). Therefore, on one hand, CaM activation causes feedback inhibition on CNGC activities and prevents a rapid increase of intracellular Ca^2+^ concentrations. On the other hand, CDPK activation (Yoon et al. 1999) leads to the phosphorylation of downstream target proteins such as Rboh (Kobayashi et al. 2006; Suzuki et al. 2011). The WRKY protein is one of the substrates of the mitogen-activated protein (MAP) kinase signalling cascade reaction (Mao et al. 2011; Zhou et al. 2015). Therefore, the WRKY transcription factor can be inferred to activate WRKY regulatory genes, especially defence-related genes. Comprehensive analyses showed that certain defence-response reactions occur in host plants at the initial stage of induction by the endophytic fungi.

### Key tanshinone synthesis-related genes and pathways

**Figure 4.**
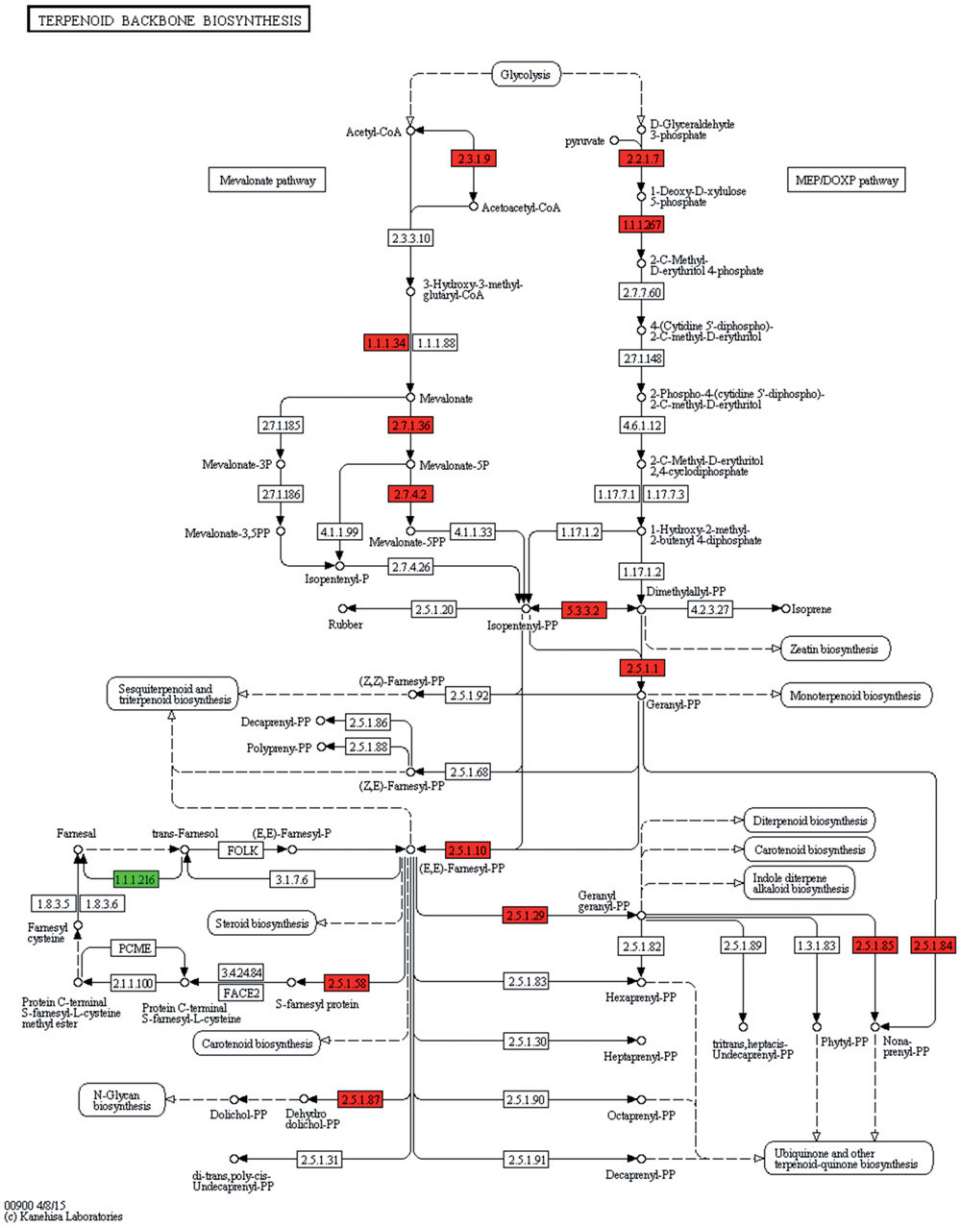
Terpenoid backbone biosynthesis. The number in the box represents the number of the enzyme (EC number). The red and green boxes represent the up-regulated and down-regulated genes, respectively. The differentially expressed enzyme genes in Figure 4 correspond to the genes in Table 5 (up-regulated genes: AACT, HMGR, MK, PMK, DXS, DXR, IDI, GPPS, FDPS, GGPPS, SPS, SDS, DHDDS, RER2, SRT1, FNTB; down-regulated genes: FLDH).

Among the 3713 obtained DEGs, 75 genes were annotated to the host plant *S. miltiorrhiza* (Table 4) and could be classified into seven groups: biological stimulus-response, transcription factor MYB, terpenoid synthesis, phenolic acid synthesis, cytochrome P450 and oxidation-reduction reaction and others. The plant transcription factor MYB is one of the largest transcription factor families in plants (Li and Lu 2014). They generally serve as positive regulatory factors and exert their functions in stress response and the phenylpropanoid metabolism pathway in plants. Cytochrome P450 extensively participates in plant development and metabolism regulation (Seki et al. 2011; Li et al. 2013; Wu et al. 2013). The CYP76AH1 annotated in this study is the first P450 gene in the tanshinone biosynthesis pathway (Guo et al. 2013, 2016; Ma et al. 2015). The upregulation of its expression might be associated with accumulation of tanshinone components. We speculated that under the induction by U104 endophytic fungi, the plant first developed defence response reactions to upregulate the expression levels of genes encoding lipid transfer protein-2 (LTP2) (Gomès et al. 2003; Wu et al. 2004), allergen, SMLII (Peumans and Van Damme 1995) and other stress response genes. Next, changes in genes encoding the transcription factor MYB and other related proteins were activated. Finally, the expression levels of the genes of key enzymes in the terpenoid biosynthesis pathway changed to promote the accumulation of *S. miltiorrhiza* active ingredients. The annotated key enzymes such as DXS (Kai et al. 2011; Ma et al. 2012), DXR (Wu et al. 2009), HMGR (Dai et al. 2011; Kai et al. 2011; Ma et al. 2012; Shi et al. 2014), MK, GGPPS (Kai et al. 2011; Ma et al. 2012), GPPS, KSL (Xu et al. 2015), IDI, IPII, FDPS and CPS (Cui et al. 2015; Xu et al. 2015) in *S. miltiorrhiza* were also present in the terpenoid compound biosynthesis pathway (Figure 4, Table 5). In addition, AACT and PMK in the mevalonic acid (MVA) upstream pathway also had upregulated expression. These key enzymes provided significant guidance in our studies on the terpenoid metabolic pathway. In future studies, the molecular functions of these enzymes can be studied using genetic methods such as gene silencing and gene overexpression.

**Table 4.** The unigenes of annotated to *Salvia miltiorrhiza*.

Number	Log_2_FC	NR_annotation
Biological stimulus response		
Smc38674.graph_c0	3.450957287	allergen
Smc30794.graph_c0	2.878779528	allergen
Smc7849.graph_c0	2.899264351	allergen
Smc25749.graph_c0	−6.425139311	putative major latex-like protein
Smc48982.graph_c1	2.057932379	SMLII
Smc38239.graph_c0	1.386618012	SMLII
Smc13539.graph_c0	6.105013294	lipid transfer protein 2
Smc37320.graph_c0	−1.097279161	thionin
Transcription factor		
Smc50276.graph_c0	−1	MYB-related transcription factor
Smc26006.graph_c0	3.010045188	MYB-related transcription factor
Smc48251.graph_c0	−2.750548542	MYB-related transcription factor
Smc35597.graph_c0	−2.838921149	MYB-related transcription factor
Smc38154.graph_c0	3.173322311	MYB-related transcription factor
Smc31062.graph_c0	3.155883414	MYB-related transcription factor
Terpenoid synthesis		
Smc35973.graph_c0	1.20912885	1-deoxy-d-xylulose 5-phosphate reductoisomerase
Smc51086.graph_c2	2.139469922	3-hydroxy-3-methylglutaryl-coenzyme A reductase 3
Smc42400.graph_c0	1.888631134	1-deoxy-D-xylulose 5-phosphate synthase 2
Smc18265.graph_c0	1.510624592	farnesyl diphosphate synthetase
Smc50008.graph_c0	1.972359308	kaurene synthase
Smc40957.graph_c0	3.374195449	geranyl diphosphate synthase small subunit type II.1
Smc30608.graph_c0	2.124075273	geranylgeranyl diphosphate synthase
Smc40214.graph_c0	1.233889374	isopentenyl pyrophosphate isomerase
Smc33319.graph_c0	2.54442726	1-deoxy-D-xylulose 5-phosphate synthase
Smc16495.graph_c0	3.583347988	isopentenyl diphosphate isomerase
Smc43553.graph_c0	3.793911331	copalyl diphosphate synthase
Smc37513.graph_c0	1.223770267	mevalonate kinase
Phenolic acid synthesis		
Smc40869.graph_c0	1.633073555	4-coumarate:coenzyme A ligase 2
Smc41710.graph_c0	1.706003738	4-coumarate:coenzyme A ligase 3
Smc39029.graph_c0	1.576414099	4-coumarate:coenzyme A ligase 7
Smc8282.graph_c0	1.992876542	cinnamate 4-hydroxylase
Smc40268.graph_c0	1.92221871	phenylalanine ammonia-lyase
Smc50941.graph_c0	−1.085664845	4-hydroxyphenylpyruvate dioxygenase
Smc36954.graph_c0	1.367461941	chorismate mutase
Smc43075.graph_c0	2.028634685	caffeoyl CoA O-methyltransferase
Cytochrome P450		
Smc42658.graph_c0	2.210418615	cytochrome P450
Smc26672.graph_c0	3.412624754	cytochrome P450
Smc8191.graph_c0	1.772687688	cytochrome P450
Smc26204.graph_c0	Inf	cytochrome P450
Smc43180.graph_c0	2.932599323	cytochrome P450
Smc49533.graph_c0	3.108274716	cytochrome P450
Smc31380.graph_c0	3.05272908	cytochrome P450
Smc8774.graph_c0	2.220826344	cytochrome P450
Smc50907.graph_c0	1.756411374	cytochrome P450
Smc45086.graph_c0	1.873130983	cytochrome P450 CYP707A102
Smc39573.graph_c0	1.88039929	cytochrome P450 CYP714A25
Smc41055.graph_c0	3.821823771	cytochrome P450 CYP714G13
Smc38993.graph_c0	1.918135719	cytochrome P450 CYP714G14
Smc44214.graph_c0	1.454847796	cytochrome P450 CYP716A89
Smc34207.graph_c0	−1.006910715	cytochrome P450 CYP716D25
Smc42664.graph_c0	1.225136143	cytochrome P450 CYP71AH15
Smc49591.graph_c0	−1.469881657	cytochrome P450 CYP72A326
Smc49732.graph_c1	1.193311904	cytochrome P450 CYP72A327
Smc50931.graph_c1	1.195404255	cytochrome P450 CYP72A327
Smc47009.graph_c0	4.143286281	cytochrome P450 CYP72A329
Smc48172.graph_c0	1.26343305	cytochrome P450 CYP736A122
Smc48884.graph_c0	1.912362769	cytochrome P450 CYP76AH1
Smc48295.graph_c0	2.41357928	cytochrome P450 CYP76AK2
Smc51456.graph_c0	2.874858511	cytochrome P450 CYP76AK3
Smc42779.graph_c0	3.439883771	cytochrome P450 CYP76AK3
Smc40217.graph_c0	1.671911638	cytochrome P450 CYP76S7
Smc13526.graph_c0	2.666006882	cytochrome P450 CYP78A113
Smc52626.graph_c0	2.270562827	cytochrome P450 CYP84A60
Smc31407.graph_c0	3.34375109	cytochrome P450 CYP92A73
Smc43546.graph_c0	3.130762483	cytochrome P450 CYP92B29
Smc48996.graph_c0	1.569092828	cytochrome P450 CYP94A48
Smc47805.graph_c0	1.497286229	cytochrome P450 CYP98A75
Smc48884.graph_c1	2.779772466	ferruginol synthase；cytochrome P450 76AH1
Oxidation reduction reaction		
Smc29807.graph_c0	−6.138087348	putative aldo/keto reductase 1
Smc43430.graph_c1	−2.36823321	putative aldo/keto reductase 1
Smc34682.graph_c0	3.891064535	alcohol dehydrogenase
Smc47960.graph_c0	2.505069973	PPO
Others		
Smc52705.graph_c0	1.518769884	ptoteinase inhibitor 2
Smc43159.graph_c0	−1.190930207	glycosyl hydrolase family-like protein
Smc40337.graph_c0	1.253730717	acetoacetyl-coenzyme A thiolase
Smc37957.graph_c1	−3.517758658	metallothionein 1

**Table 5.** The differentially expressed genes in terpenoid biosynthesis pathway.

EC number	Definition
2.3.1.9	acetyl-CoA C-acetyltransferase (AACT)
1.1.1.34	hydroxymethylglutaryl-CoA reductase (HMGR)
2.7.1.36	mevalonate kinase (MK)
2.7.4.2	phosphomevalonate kinase (PMK)
2.2.1.7	1-deoxy-D-xylulose-5-phosphate synthase (DXS)
1.1.1.267	1-deoxy-D-xylulose-5-phosphate reductoisomerase (DXR)
5.3.3.2	isopentenyl-diphosphate Delta-isomerase (IDI)
2.5.1.1	geranyl diphosphate synthase (GPPS)
2.5.1.10	farnesyl diphosphate synthase (FDPS)
2.5.1.29	geranylgeranyl diphosphate synthase, type II (GGPPS)
2.5.1.84/2.5.1.85	all-*trans*-nonaprenyl-diphosphate synthase (SPS, SDS)
2.5.1.87	di-*trans*, poly-*cis*-polyprenyl diphosphate synthase (DHDDS, RER2, SRT1)
2.5.1.58	protein farnesyltransferase subunit beta (FNTB)
1.1.1.216	NAD^+^-dependent farnesol dehydrogenase (FLDH)

## Discussion

By implementing RNA-Seq in host plants, all involved defence response DEGs were exhibited, as well as vital DEGs that promoted the accumulation of active ingredients after induction of endophyte fungi. The large amount of data generated in this study provides a powerful platform for functional and molecular studies of future interactions between host plants and their endophytic fungi. In these DEGs, CNGC, CDPK, Rboh, CaM, MAP2K1/MEK1, WRKY33, SUGT1/SGT1 and HSP90A/htpG are the DEGs that involved in biological response stimulation, in which WRKY33 belongs to the WRKY gene family, one of the largest family of plant transcription factors currently studied (Suttipanta et al. 2011; Phukan et al. 2016; Chen et al. 2017). In recent years, accumulating evidence indicates that WRKY transcription factors are not only resistant to plants, but also in plant secondary metabolism regulation (Suttipanta et al. 2011; Phukan et al. 2016; Chen et al. 2017). For example, SmWRKY2 could respond to the induction of MeJA and improve tanshinone production after the induction of *S. miltiorrhiza* using MeJA, indicating that SmWRKY2 may be involved in stress-regulated processes (Deng et al. 2019). SmWRKY1 can respond to the induction of salicylic acid (SA), methyl jasmonate (MeJA) and nitric oxide (NO), and improve the yield of tanshinone by positively regulating SmDXR expression (Cao et al. 2018). Overexpression of NtWRKY50 upregulated the expression level of related defence genes and increased tobacco resistance to *Ralstonia solanacearum* (Liu et al. 2017). Therefore, we hypothesized that the up-regulated expression of WRKY33 may be a key gene for regulating tanshinone production in response to fungal induction in plants.

In this study, seven key enzymes were up-regulated to varying degrees in the upstream of the tanshinone biosynthesis pathway: MEP pathway (DXS, DXS2, DXR), MVA pathway (AACT, MK, PMK, HMGR3). Previous studies have shown that the biosynthesis of tanshinone is mainly through the MEP pathway whereas our study found that key genes in the upstream pathway of MVA showed significant differences (FC > 6) after U104 endophytic fungi induction. Therefore, we speculate that the upstream pathway of MVA may play a key role in the synthesis of tanshinone precursors in the *S. miltiorrhiza* seedlings induced by endophytic fungi and the specific regulatory mechanisms remain to be further explored.

Induced by endophytic fungi, the host plant can respond to the fungus and increase the yield of tanshinone by regulating the genes involved in the tanshinone synthesis pathway. Our work reported RNA-seq of *S. miltiorrhiza* by endophytic fungi. In addition, some studies have compared RNA-seq of *S. miltiorrhiza* by other elicitors such as methyl jasmonate (MeJA) and yeast extract (YE). MeJA and YE responsive genes related to tanshinones and phenolic acids biosynthesis. Compared to MeJA, YE had a more significant effect on genes involved in biosynthesis of tanshinone. The expression patterns of genes involved in phenolic acid biosynthesis pathways were diverse. PAL, C4H, 4CL, TAT, HPPR, RAS and CYP98A14 were all induced by MeJA. Nevertheless, YE didn’t show any clear effect on these genes. It was also consistent with change of active ingredients contents after treatment by these two elicitors, in which the content of tanshinones in hairy root could be induced by these two elicitors, but to phenolic acid, the contents could only be induced by MeJA, not by YE (Zhou et al. 2017). However, the mechanism of interaction between endophytic fungi and host still needs a lot of research and repeated verification owing to the lack of studies on the interaction between fungi and *S. miltiorrhiza* and studies on the metabolic pathway of tanshinone in endophytic fungi with the same active ingredients in the host.
